# Browning capabilities of human primary adipose-derived stromal cells compared to SGBS cells

**DOI:** 10.1038/s41598-020-64369-7

**Published:** 2020-06-15

**Authors:** D. Halbgebauer, M. Dahlhaus, M. Wabitsch, P. Fischer-Posovszky, D. Tews

**Affiliations:** Division of Pediatric Endocrinology and Diabetes, Department of Pediatric and Adolescent Medicine, Ulm University Medical Center, Ulm, Germany

**Keywords:** Obesity, Molecular medicine

**arising from**: C. R. Yeo et al.; *Scientific Reports* 10.1038/s41598-017-04369-2 (2017).

## Introduction

Induction of a brown adipocyte differentiation program in white adipocytes (so-called “browning”) by pharmacological agents leads to improved energy metabolism thus providing a therapeutic option to treat obesity. In a recent study, Yeo *et al*. claimed that the Simpson-Golabi-Behmel syndrome (SGBS) cell strain is superior in browning compared to primary adipose-derived stromal cells (hASCs); however, different media compositions were used for the different cell types potentially hampering interpretation of results. Comparing both cell types under equal conditions we demonstrate that both, SGBS and hASCs, differentiate into brown-like adipocytes, but with differences regarding UCP1 expression and mitochondrial content underlining that media conditions strongly influence the adipocyte phenotype. This should be considered in future studies.

In contrast to white adipose tissue (WAT), the primary energy storage organ of the body, brown adipose tissue (BAT) is able to metabolize lipids and glucose to produce heat in order to defend body temperature against cold^1^.

Upon chronic cold exposure, white adipose tissue (WAT) can partially convert into thermogenic, beige adipose tissue, which shares main similarities with classical brown adipose tissue (BAT). This conversion of WAT to beige fat, often called “browning” or “beigeing” is associated with improved glucose and lipid metabolism in mice^2^. In humans, data on WAT browning is scarce^3^ but long-term cold exposure of human subjects is associated with increased BAT activity and a decrease in body fat mass^4,5^. Thus, activation of BAT has frequently been suggested as a therapeutic option to prevent or to treat obesity^6^. While BAT and beige fat develops in clear distinct depots in mice, the discrimination between brown and beige adipocytes is less defined in humans^7^.

To better understand the molecular mechanism of WAT browning, *in vitro* cell models of murine and human origin have been frequently used, including primary adipose-derived stem cells, iPS cells, hMADS and SGBS cells^8–10^. In a recent paper published by Yeo *et al*. in this journal, SGBS cells were compared to human primary adipose-derived stromal cells (hASCs) in terms of adipocyte browning *in vitro*^11^. The authors showed that SGBS adipocytes had more UCP1 mRNA expression compared to primary cells. Additionally, SGBS adipocytes showed higher respiration rates compared to primary hASCs. Based on these findings, they claimed that SGBS cells have a higher capacity to differentiate into brown-like adipocytes and concluded that SGBS cells represent a model for human brown adipocytes. Obviously however, SGBS cells in their study were apparently better differentiated than primary cells as judged by differences in lipid content and expression of adipogenic marker genes. This is most likely based on differences in media conditions used in this study.

SGBS cells were derived from subcutaneous white adipose tissue of an infant suffering from the Simpson-Golabi-Behmel syndrome (SGBS) and were established as cell strain by our lab^12^. Although the cells are not transformed or immortalized, they retain their capacity to differentiate into adipocytes *in vitro* for more than 50 generations^13^. The molecular reason for this is currently unknown. With more than 150 published articles, SGBS cells represent a valuable cell model for human adipogenesis and adipocyte biology. Yeo *et al*. recently claimed that SGBS cells have a particularly high capacity to undergo browning, whereas hASCs derived from subcutaneous white adipose tissue represent a model system to study white adipocytes^11^. Indeed, SGBS cells have been used to study *in vitro* browning of adipocytes^10,14^. In comparative studies performed so far SGBS behaved very similar to primary *in vitro* differentiated adipocytes^13^.

Therefore, we hypothesize that both SGBS as well as hASCs behave similarly regarding differentiation into brown-like adipocytes. To address this, we investigated marker gene expression of brown adipogenesis in SGBS and hASCs upon differentiation. In order to account for differences in media composition, we also compared rosiglitazone and indomethacin towards their ability to induce adipogenic differentiation, BAT marker expression and mitochondrial metabolism.

## Materials and Methods

### Ethical note

All procedures involving human subjects were approved by the ethics committee of the University of Ulm (entry number 300/16). Written informed consent was obtained from all subjects and all associated methods were conducted in accordance with approved guidelines for human experimental research.

### Cell culture

Human Simpson-Golabi-Behmel (SGBS) preadipocytes were cultured as described in the original publication^12^. Human adipose-derived stromal cells were isolated from mammary adipose tissue from n = 7 women (mean age 48 +/−16 years, mean BMI 27.4 +/−5.2 kg/m²) undergoing elective surgery using collagenase digestion (type II, Sigma-Aldrich, Munich, Germany) according to established protocols^15^.

SGBS preadipocytes and hASCs were seeded into cell culture vessels and were differentiated for 14 days into mature adipocytes using differentiation media (DMEM:F12 supplemented with 20 nM insulin, 100 nM cortisol, 25 nM dexamethasone, 250 µM IBMX, 10 µg/ml apo-transferrin, 3.3 mM biotin, 1.7 mM panthotenate) supplemented with either rosiglitazone (2 µM) or indomethacin (100 µM). After 4 days, rosiglitazone/indomethacin, IBMX and dexamethasone were omitted. Differentiation rates were determined by microscopic cell counting using a net micrometer and dividing differentiated adipocytes by total cell number.

In a different study, ASCs were isolated from paired deep neck and subcutaneous neck adipose tissue from n = 12 patients undergoing neck surgery^16^ (age 47.4 + /−18.0 years; BMI 27.3 + /−5.3 kg/m^2^), and were differentiated as described before^16^.

Triglycerides were extracted from adipocytes using hexane:isopropanol (3:1) and were dissolved in isopropanol after evaporation. Triglyceride content was determined using the Triglyceride determination kit (Merck, Darmstadt, Germany).

### Expression analysis

Extraction of total RNA, synthesis of cDNA and analysis of mRNA expression by qPCR was done as described previously^17^. Relative mRNA levels were determined by comparison to a reference gene (TF2B, SDHA) using the ddCT method. Primer sequences are available on request.

### Protein quantification and Western blot

Extraction of cellular proteins, determination of protein content and immunodetection was described before^17^.Expression of target proteins was analyzed by incubating membranes with primary antibodies (anti-UCP1 MAP6158, R&D; anti-OXPHOS ab110411, Abcam; anti-PGC1a ab54481, Abcam; anti-PLIN ab3525, Abcam; anti-TIMM23 ab116329, Abcam; hFAB rhodamine anti-GAPDH 12004168, BioRad) and HRP-conjugated secondary antibodies. ECL signals were detected using a ChemiDoc MP Imaging system (BioRad Laboratories GmbH, Munich, Germany).

### Citrate synthase assay

Citric acid synthase activity was assayed as a measure for mitochondrial content as described previously^17^.

### Functional extracellular flux analysis

Oxygen consumption was determined using a plate-based respirometer (Seahorse XFe96 Flux Analyzer, Agilent Technologies). Preparation of cells and measurement of the cellular respiration was described before^17^. Data was normalized to cell number by quantification of Janus Green incorporation^18^.

### Statistics

GraphPad Prism version 7.03 (GraphPad Software Inc., San Diego, USA) was used for statistical analysis. If not otherwise stated, data from three independent triplicate experiments were expressed as mean + /- standard error of means (SEM). For statistical comparison, Analysis of variants test (ANOVA) or t-test was used as indicated in the figure legends. A p value p < 0.05 was considered statistically significant.

## Results

### Adipogenic differentiation depends on media conditions

**Figure 1:**
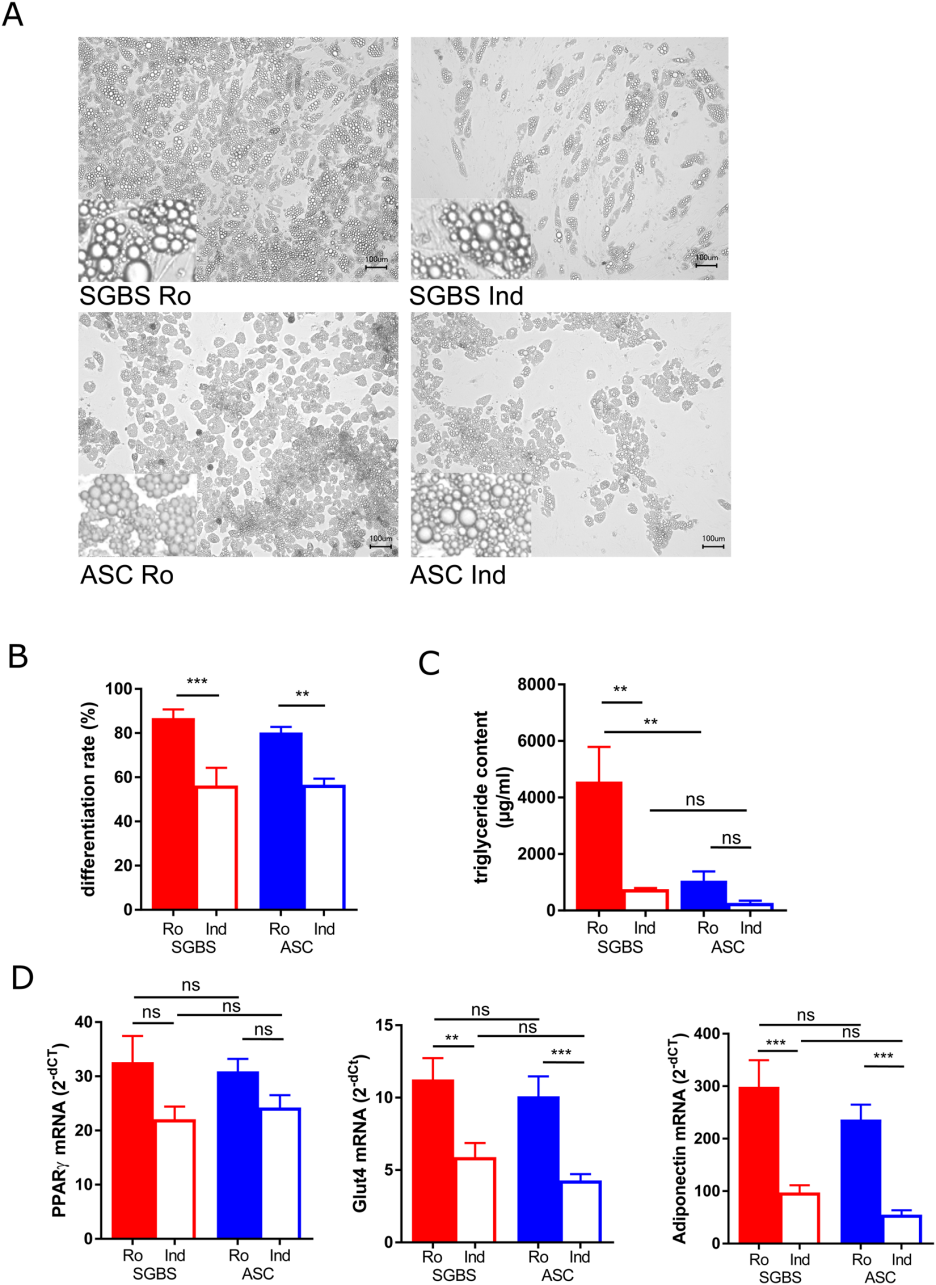
Differentiation of SGBS preadipocytes or hASCs with indomethacin or rosiglitazone. SGBS preadipocytes (n = 5 independently performed experiments) or human adipose stromal cells (hASC, n = 7 different donors) were differentiated *in vitro* using either 100 µM indomethacin (Ind) or 2 µM rosiglitazone (Ro) for 14 days, representative photomicrographs shown in (**A**) inlets 5-fold enlarged. The adipogenic differentiation rate (**B**) was determined microscopically. Triglyceride content was enzymatically determined (n = 3–4) (**C**). The expression of key adipogenic marker genes was assessed by qRT-PCR using the dCt method, TF2B was used as reference gene (**D**). Mean +SEM is shown, *p < 0.05, **p < 0.01, ***p < 0.001, ****p < 0.0001.

SGBS and hASCs derived from mammary subcutaneous adipose tissue were subjected to adipogenic differentiation in media supplemented with either rosiglitazone or indomethacin. Within 14 days, cells accumulated lipids and turned from fibroblastic into the characteristic shape of *in vitro* differentiated adipocytes. Obviously, fewer cells were differentiated when using indomethacin in both SGBS and hASCs (Fig. 1A). This was also evident when analyzing differentiation rates (Fig. 1B). Under rosiglitazone conditions, both SGBS and hASC differentiated to a comparable extent (86.8 + /−8.7 and 80.3 + /−6.6%). Using indomethacin, differentiation rates were much lower in general, but also comparable between both cell types (56.3 + /−17.9 and 56.6 + /−7.1%). These results were also reflected by comparable expression rates of the adipogenic marker genes PPARg (peroxisome proliferator-activated receptor gamma), adiponectin, and GLUT4 (glucose transporter 4, Fig. 1D). Interestingly, SGBS cells seemed to have larger lipid droplets compared to hASCs (Fig. 1A), which was also reflected by increased triglyceride content (Fig. 1C).

### Adipocyte browning capacity is different in SGBS cells and hASCs

**Figure 2:**
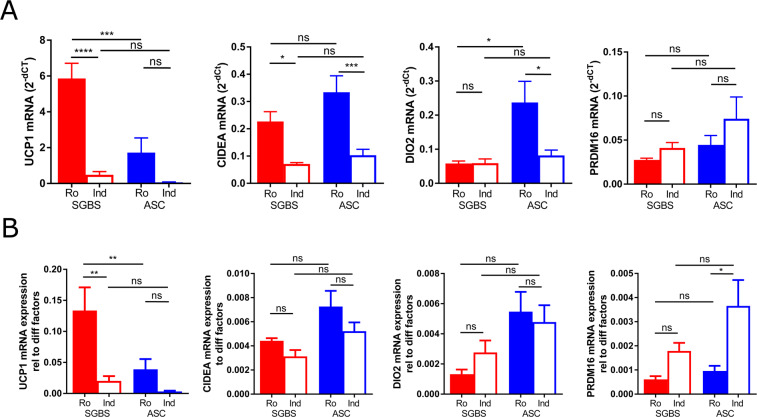
Expression of BAT-associated genes in SGBS or hASCs differentiated with indomethacin or rosiglitazone. SGBS preadipocytes (n = 5 independently performed experiments) or human adipose stromal cells (hASC, n = 7 different donors) were differentiated *in vitro* using either 100 µM indomethacin (indo) or 2 µM rosiglitazone (rosi) for 14 days. Total RNA was isolated and the expression of UCP1, CPT1B, PGC1a and DIO2 was determined by qRT-PCR using TF2B as reference (**A**). Data was also normalized using the geometric mean of adipogenic marker genes in Fig. 1D as reference (**B**). Mean +SEM is shown, *p < 0.05, **p < 0.01, ***p < 0.001, ****p < 0.0001.

Subsequently, we assessed the expression of BAT-related genes in SGBS and hASCs under the chosen conditions. UCP1 was higher expressed in adipocytes differentiated with rosi compared to indomethacin, where UCP1 expression was hardly detectable (Fig. 2A). Interestingly, SGBS cells showed stronger UCP1 expression compared to hASCs when differentiated with rosiglitazone. CIDEA was equally expressed between SGBS and hASCs, being approximately 3-fold higher expressed in rosi compared to indo conditions. DIO2 (deiodinase 2) was induced by rosiglitazone compared to indo in hASCs only. Surprisingly, PRDM16 was not induced by rosiglitazone in both cell types (Fig. 2A). To account for differences in differentiation rates between cell types and individual samples, expression data of BAT-associated genes were normalized to the geometric mean of the CT values of PPARg, adiponectin, and GLUT-4. Interestingly, UCP1 mRNA as well as protein expression was higher in SGBS compared to hASCs even after correction for differentiation, suggesting higher propensity to browning in SGBS cells. (Fig. 2B).

**Figure 3:**
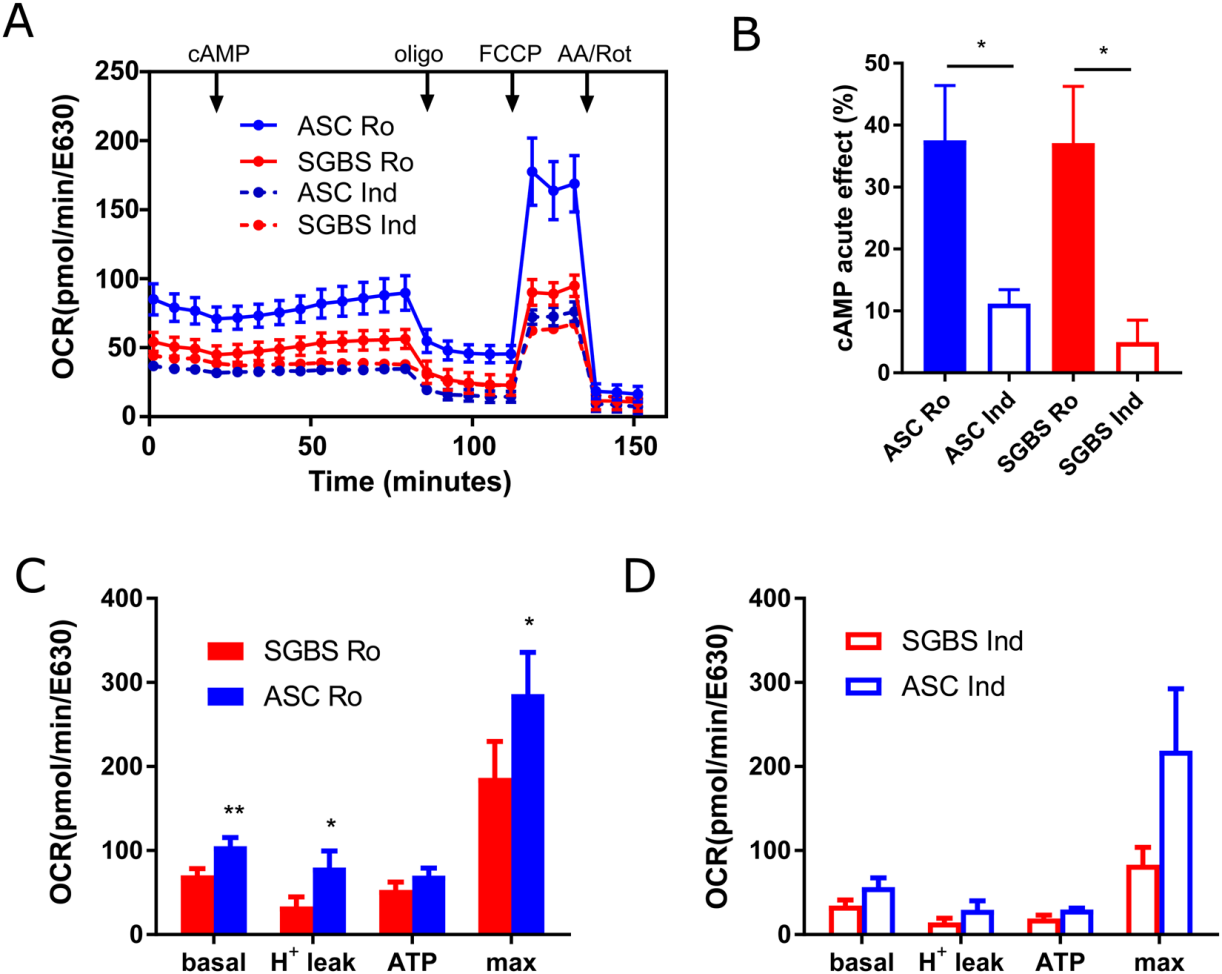
Mitochondrial activity of SGBS and hASC adipocytes. SGBS and hASCs (n = 4 each) were differentiated *in vitro* using either 100 µM indomethacin (Ind) or 2 µM rosiglitazone (Ro) for 14 days and subjected to respiration measurements using a plate-based respirometer (Seahorse XFe96, Agilent, **A**). Cells were measured in a 3 min mix – 3 min measure scheme. After 3 basal measurements, 0.5 mM dibutyryl-cAMP was injected to induce UCP1 activity by lipolysis. Oligomycin (2 µM) was added to determine ATP and proton leak-dependent respiration. Full uncoupling of mitochondrial ETC was achieved by addition of 4 µM FCCP. Finally, ETC was completely blocked by antimycin A and rotenone (1.5 µM each). Assay was performed in medium containing 1% BSA. Relative induction of respiration by cAMP was calculated as difference between first and second injection (**B**). Basal and proton leak respiration in cells differentiated with rosiglitazone (**C**) or indomethacin (**D**) was calculated from the oxygen consumption rate (OCR) plots. Data are shown as mean +SEM is shown, *p < 0.05, **p < 0.01.

To identify differences in mitochondrial metabolism, we performed respiration analyses using a plate-based respirometer (Fig. 3A). Upon differentiation with rosiglitazone, basal, proton leak, and maximal respiration was significantly higher in hASCs compared to SGBS adipocytes (Fig. 3C). This effect was also observed, although not significantly different, in indomethacin-differentiated cells (Fig. 3D). Of note, cAMP-driven increase in respiration was higher in cells differentiated with rosiglitazone, but there was no difference between hASCs and SGBS (Fig. 3B), suggesting that UCP1 activation by the release of free fatty acids was not different between cell types. Respiration according to ATP production was not different between SGBS and hASC adipocytes, indicating that differences in basal respiration are based on proton leak only.

### Mitochondrial density is increased in hASC compared to SGBS cells

**Figure 4:**
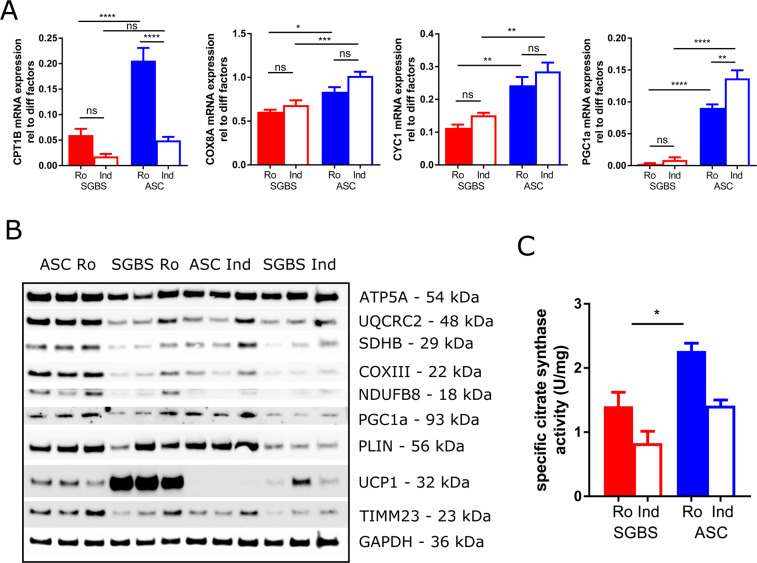
Mitochondrial content of differentiated SGBS and hASC adipocytes. SGBS preadipocytes (n = 5) or human adipose stromal cells (hASC, n = 7) were differentiated *in vitro* using either indomethacin (indo) or rosiglitazone (rosi) for 14 days. Expression of mitochondrial genes were analyzed using qRT-PCR (**A**) and on protein level (**B**). Activity of citrate synthase in cell lysates (n = 3 each) was assayed as a marker for mitochondrial content (**C**). Mean +SEM is shown, **p < 0.01, ***p < 0.001, ****p < 0.0001.

The overall higher mitochondrial metabolism in hASC suggests that the amount of mitochondria is higher in these cells compared to SGBS cells. Indeed, mRNA expression of the mitochondrial marker genes CPT1B, COX8A, and CYC1 was significantly higher in hASC compared to SGBS adipocytes when differentiated with rosiglitazone (Fig. 4A). Under indomethacin conditions, COX8A and CYC1 expression was elevated as well. In both conditions, the transcription factor essential for mitochondrial biogenesis, PGC1a, was highly increased in hASCs compared to SGBS, indicating enlarged mitochondrial content in hASC adipocytes. Protein markers of the mitochondrial electron transport chain (ETC) and the marker of the inner mitochondrial membrane TIMM23 were elevated in hASCs compared to SGBS when differentiated with rosi (Fig. 4B). Interestingly, PGC1a, the key factor for mitochondrial biogenesis, was also higher expressed in hASCs upon rosi treatment compared to SGBS. In concordance with this, activity of citrate acid synthase was also increased in rosi-differentiated hASC adipocytes compared to SGBS (Fig. 4C).

### Browning capacity is dependent on adipose depot/microenvironment

**Figure 5:**
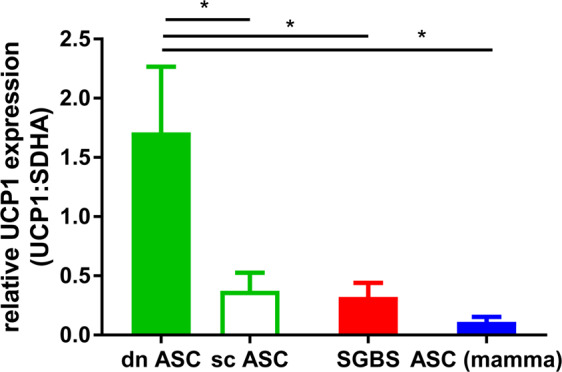
UCP1 expression in SGBS adipocytes compared to differentiated adipocytes derived from deep neck tissue. SGBS preadipocytes (n = 11) and hASCs from human deep neck (dn, n = 12) and subcutaneous neck (sc, n = 12) and mammary adipose tissue (n = 7) were differentiated into adipocytes as outlined in Fig. 1. UCP1 expression was analyzed by qRT-PCR using SDHA as reference gene. Mean +SEM is shown, *p < 0.05.

Both SGBS cells as well as hASCs derive from subcutaneous white adipose tissue. In order to appreciate their browning capacity, we compared SGBS and hASCs to progenitor cells isolated from subcutaneous and deep neck adipose tissue obtained from neck surgeries^16^, all differentiated in the presence of rosiglitazone. Clearly, cells derived from the deep neck depot displayed the strongest UCP1 expression (approx. 6-fold higher compared to subcutaneous cells), indicating highest ability to differentiate into brown adipocytes (Fig. 5). All the other cells derived from different subcutaneous adipose tissue had comparable UCP1 expression. Of note, we could not detect differences in UCP1 expression between males and females (Supplemental Fig. 2).

## Discussion

Cell culture models of human adipocyte progenitor cells are a valuable tool to evaluate the impact of genetic modulation or pharmacological treatment on induction of a white-to-brown shift in adipogenesis. Intrinsic differences in cell models (*e.g*. genetic background, immortalization etc.) as well as differences in adipogenic inducers might have strong effects on the expected outcome of the experiments. Thus, comparison of cell models and differentiation cocktails is needed to understand their impact on the regulation of adipocyte browning.

The most commonly used agents to differentiate adipocyte progenitor cells into adipocytes are thiazolidinediones (TZDs) such as troglitazone or rosiglitazone, which directly target PPARg, the key mediator of adipogenesis. As a „side-effect“ of strong PPARg induction however, thiazolidinediones are able to induce a white-to-brown phenotype switch (e.g. increased UCP1 expression) in adipocytes^19^. Mechanistically, TZDs act by binding to and activating PPARg and PPAR-response elements (PPREs) on the promoter and/or enhancer of brown fat-selective genes^20^. This mechanism of action, however, cannot be solely responsible for white-to-brown transitions because PPARg is highly and equally expressed in both brown and white adipocytes and is essential for adipogenesis in both cell types^21,22^. Of note, ectopic expression of PPARg does not induce white-to-brown transition^23^.

In earlier differentiation protocols, indomethacin, a non-steroidal anti-inflammatory drug, was frequently used as an inducer of adipogenesis^24^. It acts by inhibition of cyclooxygenases 1 (COX1) and -2 (COX2), and also induces PPARg, although activation is far lower compared to rosiglitazone^25^. As a consequence, indomethacin does not induce UCP1 expression in white adipose tissue^26^.

Interestingly, also other specific PPARg agonists fail to induce UCP1 upon adipogenic differentiation^19^, suggesting that UCP1 induction by rosiglitazone is not mediated by PPARg activation. It has been previously shown that rosiglitazone induces a white-to-brown transition in murine preadipocytes via stabilization of PRDM16, a coactivator of PPARg^19^. Whether or not PRDM16 stabilization plays a major role in human cells is not well understood. In our study, PRDM16 expression levels were low and we did not observe differences between SGBS and hASCs, which however does not exclude a role of PRDM16 in this context. Our data are in line with this literature as adipogenic differentiation was lower after indomethacin treatment in both cell models in comparison with rosiglitazone treatment. Moreover, UCP1 expression was far less induced, indicating rosiglitazone as the more potent browning inducer in this setting. Here we want to point out the importance in choosing the right differentiation condition in experiments regarding adipose browning. One should consider that rosiglitazone is a potent inducer of browning^19^ – thus, expected targeted effects might be covered by rosiglitazone action. Even more importantly, different cell types should be differentiated under the exact same conditions as they interfere with both differentiation itself, but also with the process of browning. In contrast to the earlier publication by Yeo *et al*. we could demonstrate that – if differentiated under equal conditions – SGBS and hASCs do not differ in expression of key adipogenic differentiation marker genes (e.g. PPARg, adiponectin, and GLUT-4). Thus, the absence of UCP1 mRNA expression as well as low respiration rates in hASCs in the previous paper were most likely caused by indomethacin used as an inducing agent leading to a low differentiation rate.

Although there was no significant difference in adipogenic differentiation, our data indicate that hASC and SGBS cells differ in terms of UCP1 inducibility and mitochondrial content. UCP1 expression was higher in SGBS cells compared to hASCs, however other BAT markers such as CIDEA, DIO2 and PRDM16 were either higher in hASCs or equal between the two cell types. This indicates that SGBS and primary hASCs behave differently in response to rosiglitazone treatment. Moreover, mitochondrial biogenesis is apparently higher in primary hASCs as given by the elevated mRNA levels of PGC1a and protein expression of OXPHOS components. Higher mitochondrial content in hASCs might also explain elevated basal and maximal respiration levels compared to SGBS cells.

SGBS cells were isolated from a 3-month-old infant, this might explain their capacity to stronger UCP1 induction. During ageing there is a gradually loss of BAT in humans^27^, and human studies also suggest that BAT activity is reduced in older subjects^28^. Additionally, mouse studies suggest that browning capacity decreases with ageing^29^. Variances in mitochondrial biogenesis might be caused by either to different origin of adipose tissue (subcutaneous versus mammary) or the underlying disease of the donor (SGBS). In-depth sequencing of the SGBS cells might identify the cause for reduced mitochondrial content.

Both SGBS cells and hASCs showed the capacity to differentiate into UCP1 expressing adipocytes and might reflect the situation occurring in white adipose tissue *in vivo*. Compared to progenitor cells from the deep neck depot^16^, a site where brown adipocytes can be found in humans, the induction of UCP1 expression is lower in SGBS and hASCs and is comparable to subcutaneous-derived cells from the neck. It has been shown by us and others that cells from the deep neck depot have stronger ability to differentiate into brown adipocytes *in vitro* compared to those isolated from subcutaneous tissues^16,30,31^. This suggests that browning capacity is dependent on the given adipose tissue niche or that a certain progenitor cell exists which has the machinery to differentiate into brown-like adipocytes.

In summary, we provide evidence that both SGBS cells and hASCs are both able to differentiate into UCP1-positive adipocytes, using rosiglitazone as adipogenic inducer. Comparing these cells to cells originating from a brown adipose location it is obvious that the respective depot is an important driver of brown adipogenesis.

## Supplementary information

Supplementary information.

## Data Availability

The datasets generated during and/or analyzed during the current study are available from the corresponding author on reasonable request.
