# Supplementary material for: Browning capabilities of human primary adipose-derived stromal cells compared to SGBS cells
Source: Sci Rep. 2020 Jun 15;10:9632. doi: 10.1038/s41598-020-64369-7 (PMC7296016; doi:10.1038/s41598-020-64369-7)
Supplement: Supplementary file 1 — Supplementary information. [file 41598_2020_64369_MOESM1_ESM.docx]

**Supplementary information**

**Browning capabilities of human primary adipose-derived stromal cells compared to SGBS cells**

Halbgebauer D, Dahlhaus M, Wabitsch M, Fischer-Posovszky P, Tews D

Division of Pediatric Endocrinology and Diabetes, Department of Pediatric and Adolescent Medicine, University Medical Center Ulm, Germany

**Supplementary figure S1**


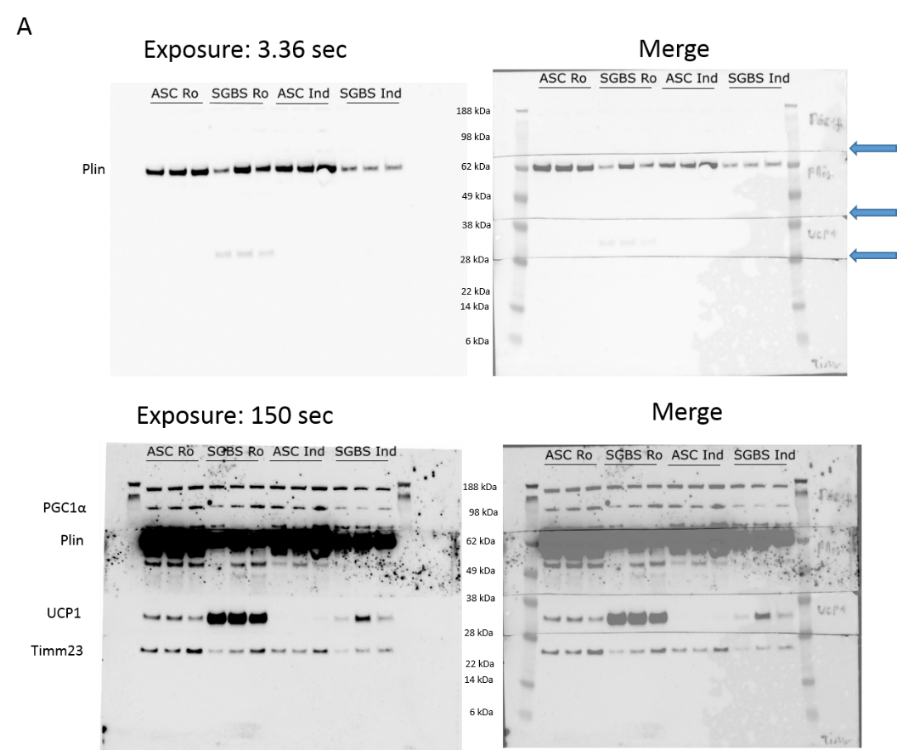


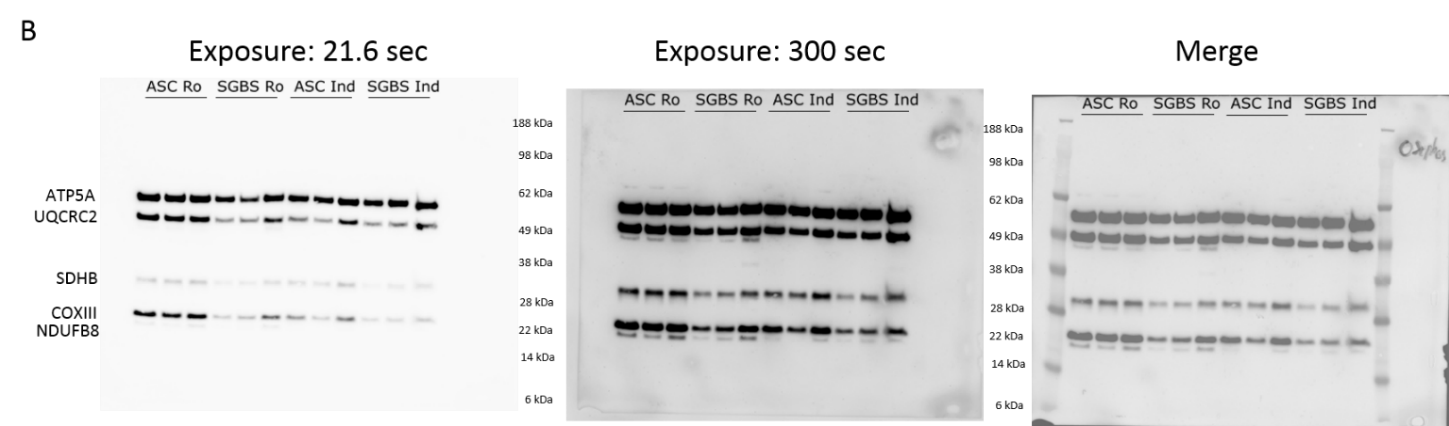


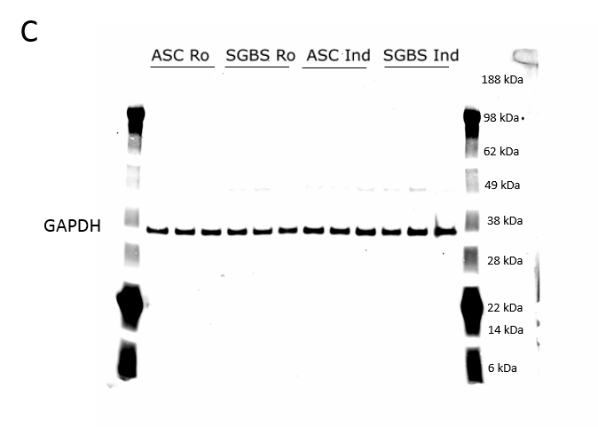


**Supplementary figure S1: Full-length blots of protein expression in ASC’s and SGBS adipocytes with different exposure times.** A: Chemiluminescent blots of Plin, PGC1α, UCP1 and Timm23 protein expression and merged with colorimetric picture to show molecular weight marker. Arrows indicate cuts on the membrane. B: Chemiluminescent blots of mitochondrial genes and merged with colorimetric picture to show molecular weight. C: Fluorescent rhodamine blot of GAPDH

**Supplementary figure S2: Differences in UCP1 expression between adipocytes differentiated from neck-derived ASCs.** ASCs from human deep neck and subcutaneous neck adipose tissue were differentiated in medium containing rosiglitazone. Data from Fig. 5 are divided into male (n=8) and female (n=4) samples. Mean +SEM is shown.
